# Supplementary material for: A defensin-like protein drives cadmium efflux and allocation in rice
Source: Nat Commun. 2018 Feb 13;9:645. doi: 10.1038/s41467-018-03088-0 (PMC5811569; doi:10.1038/s41467-018-03088-0)
Supplement: Supplementary file 1 — Supplementary Information [file 41467_2018_3088_MOESM1_ESM.pdf]

Supplementary information

Luo et al, A Defensin-like protein drives cadmium efflux and allocation in rice

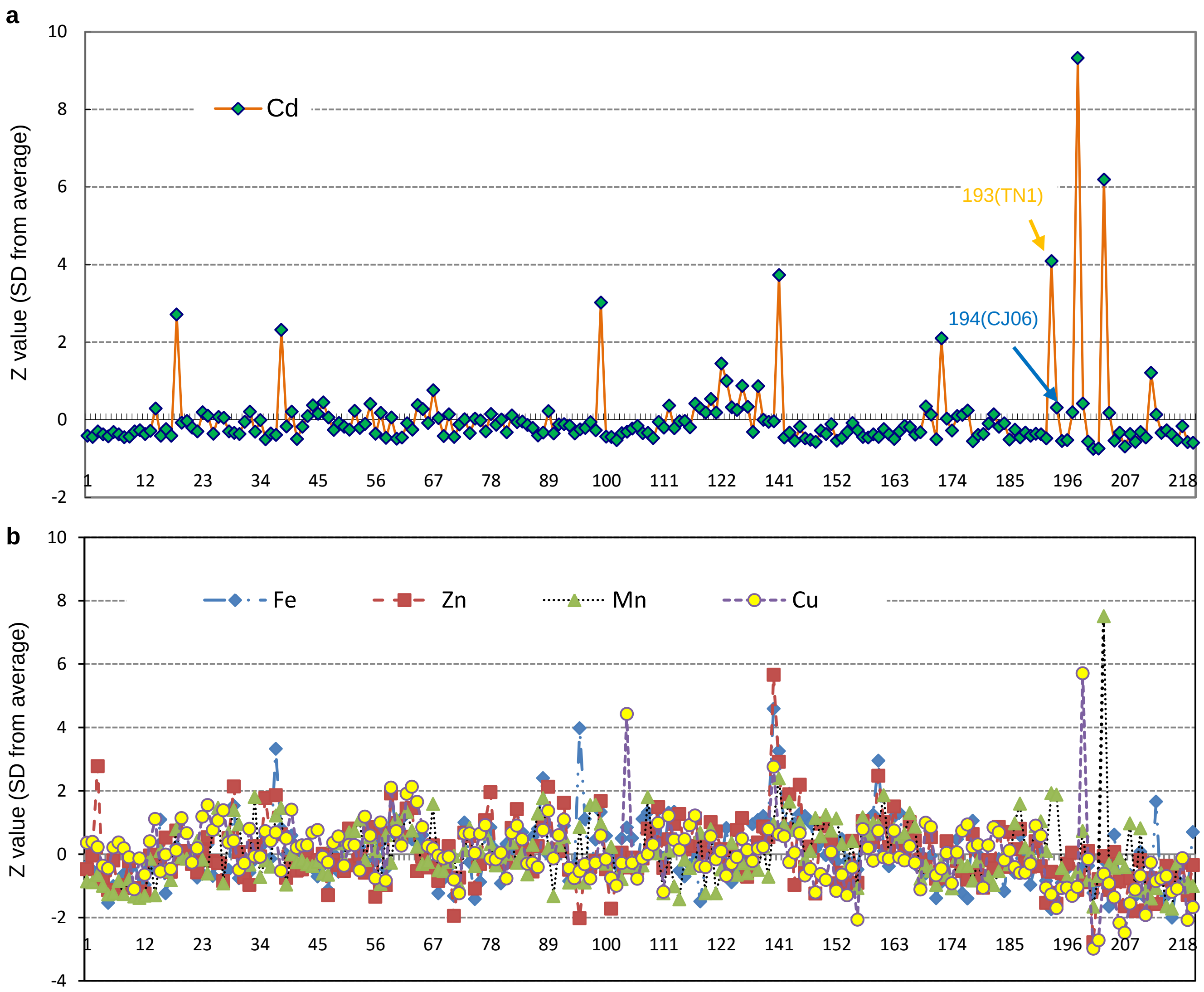

Supplementary Figure 1. Profiling of Cd (a) and Fe/Zn/Mn/Cu (b) accumulation in grains of 212 rice accessions grown in contaminated paddy field. Z value (y axis) represents SD from the average value of the whole population [Z = (individual value-population average)/population SD, Gong et al., 2004, PNAS, 101:15404-]. X axis shows the serial number (refer to Supplementary Table 1 for accession names) designed to each accession. Orange and blue arrows in (a) indicate the rice accessions used for further QTL analysis.

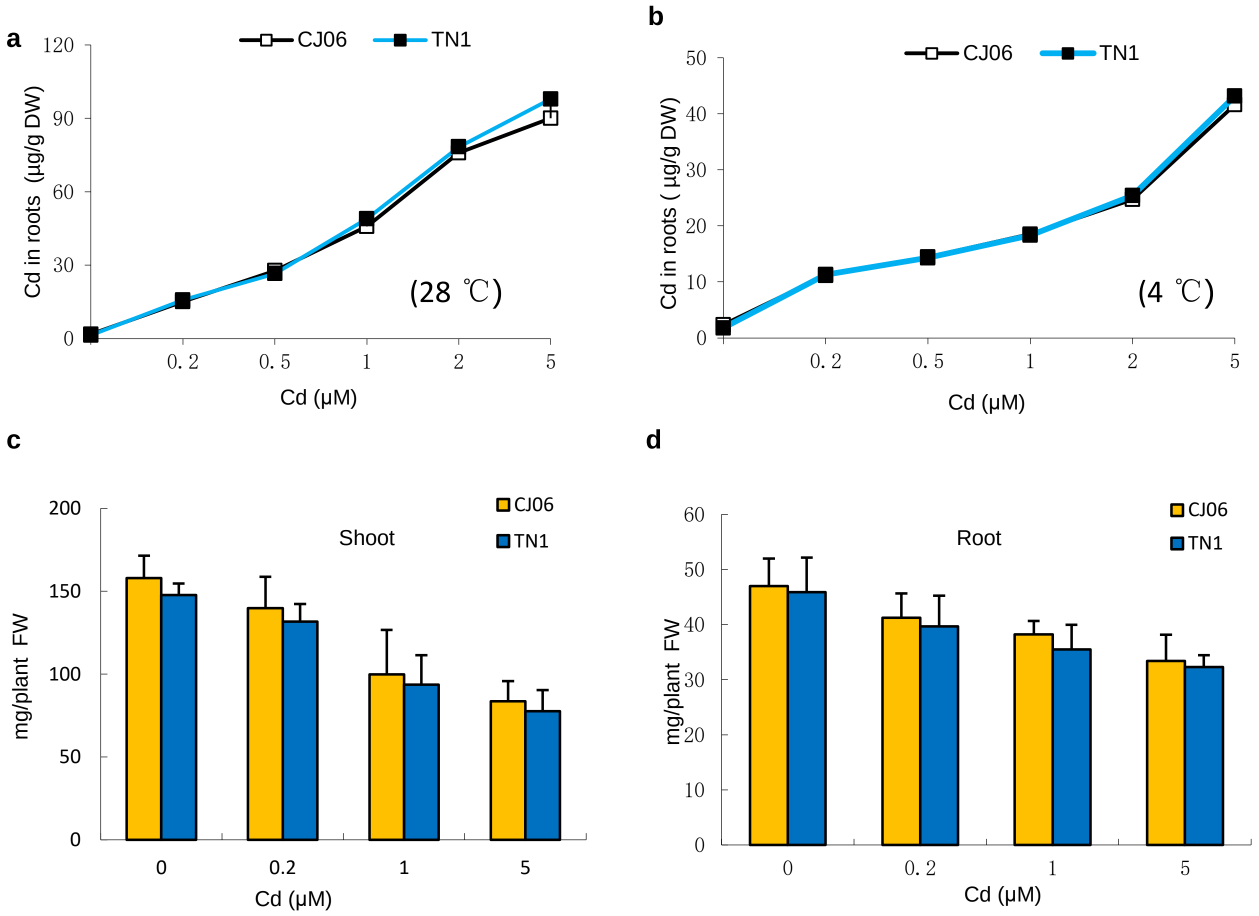

Supplementary Figure 2. Determination of Cd sensitivity and uptake. (a-b) Cd uptake assay. Two weeks old seedlings grown in hydroponics were exposed to Cd treatments for 20 minutes, at 28℃ (a) or 4℃ (b), respectively, and Cd accumulation in roots was determined. (c-d) Cd sensitivity assay. Rice seedlings were grown for 14 days in hydroponics supplemented with Cd at indicated concentrations, and then sampled to determine fresh weights (FW) of shoots (c) or roots (d). Data are mean ± SD, n = 4 in (a-d).

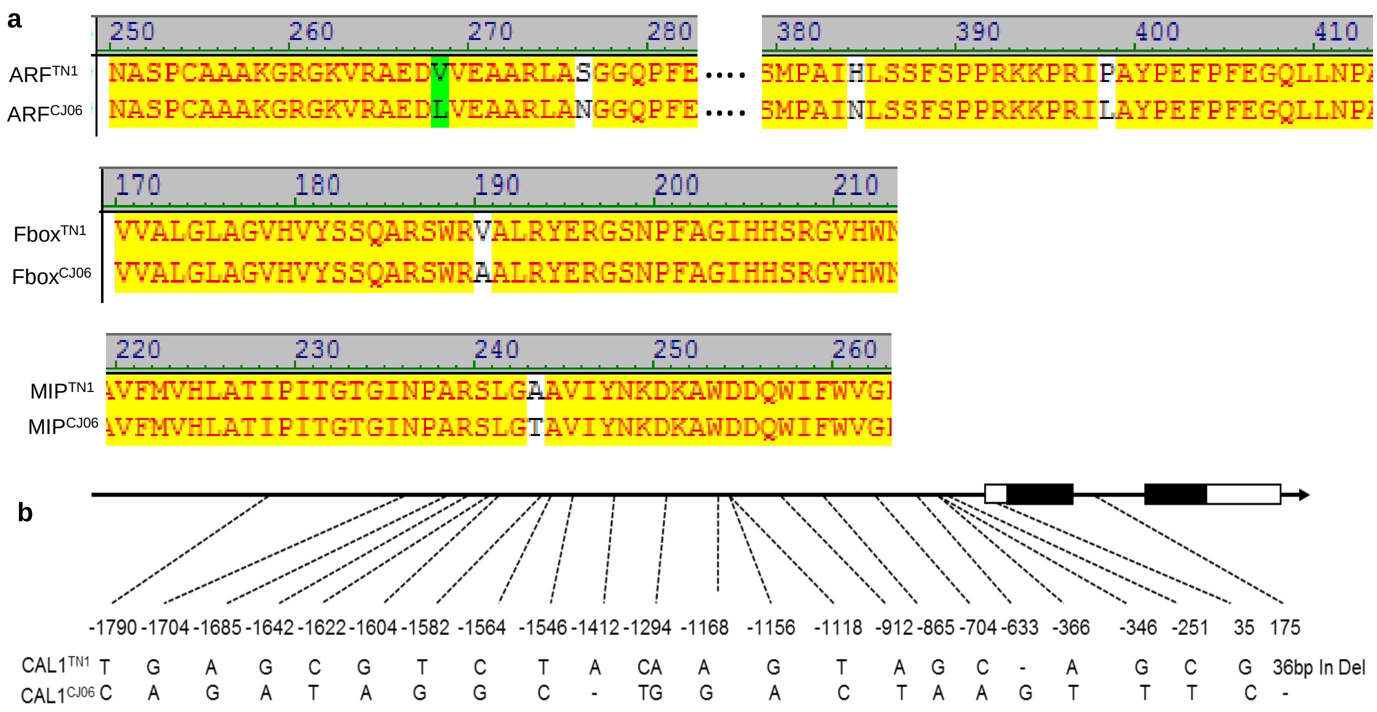

Supplementary Figure 3. Mapping-based cloning of the casual gene for *CAL1*(a) Alignment of protein sequences encoded by candidate genes *ARF* (Os02g0628600), *F-box* (Os02g0630000) and *MIP* (Os02g0629200) from the parental accessions TN1 and CJ06.(b) Natural variations in Os02g0629800 (*CAL1*) between TN1 and CJ06. Open boxes: 5'-UTR or 3'-UTR. Closed boxes: exons. The black line before 5'UTR represents the promoter region.

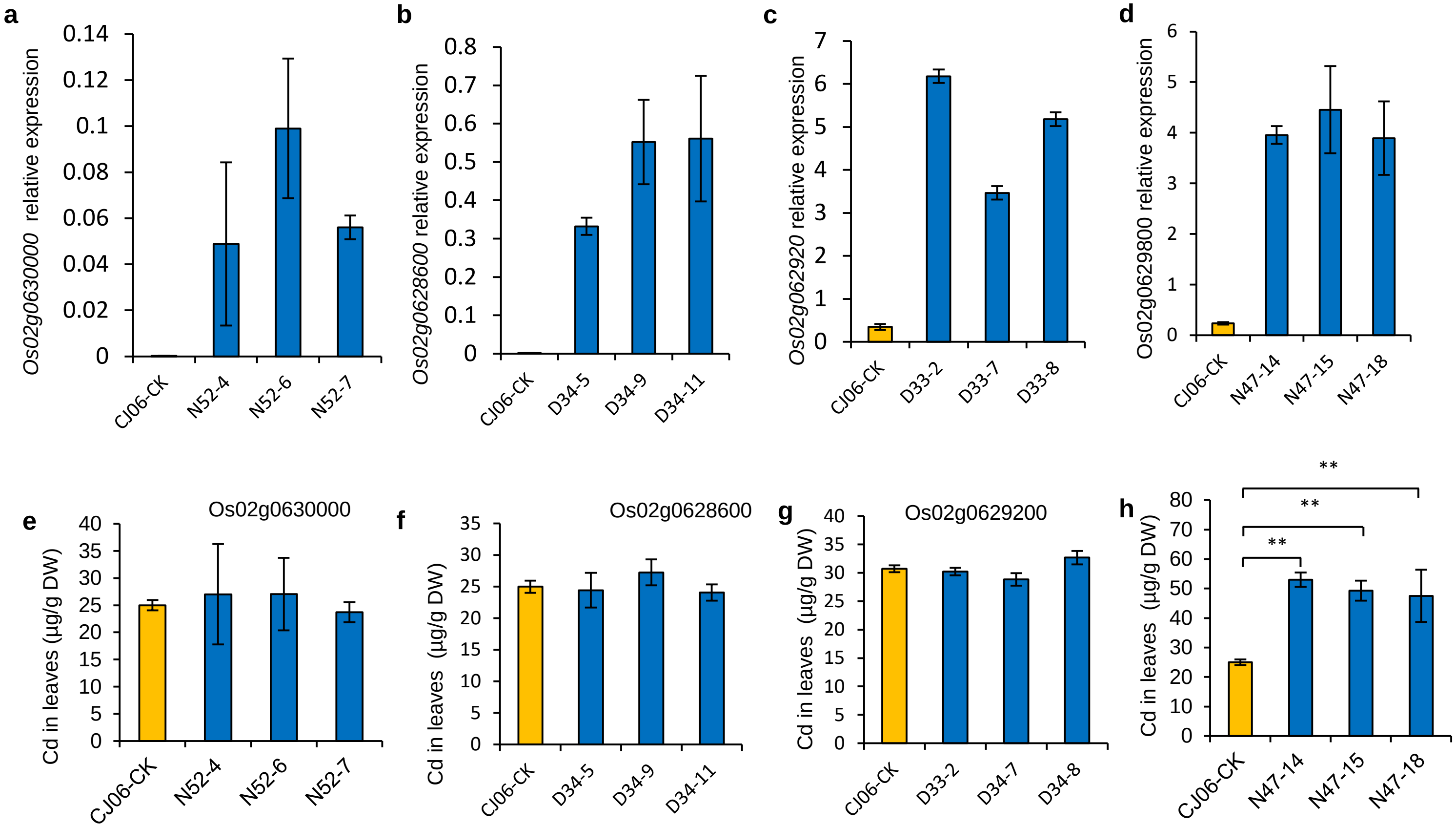

Supplementary Figure 4. Complementation test of the candidate genes. Genomic sequence of candidate genes from the over-accumulation accession TN1 were cloned and transformed into the under-accumulation accession CJ06. Transgenic seedlings harboring *Os02g0630000*^{TN1}, *Os02g0628600*^{TN1},*Os02g0629200*^{TN1} or *Os02g0629800*^{TN1} (*CAL1*) were grown in hydroponics to 2 weeks old and then exposed to 10 µM Cd treatment for 7 days. The plants were then sampled to determine expression levels of candidate genes (a, b, c, d) or Cd accumulation in leaves (e, f, g, h). CJ06-CK represents transgenic plants that did not harbor the transformation construct and were used as controls. *Actin* was used as internal controls. Data are mean ± SD, n = 4. Significant differences were determined by Student's t-test (**P < 0.01).

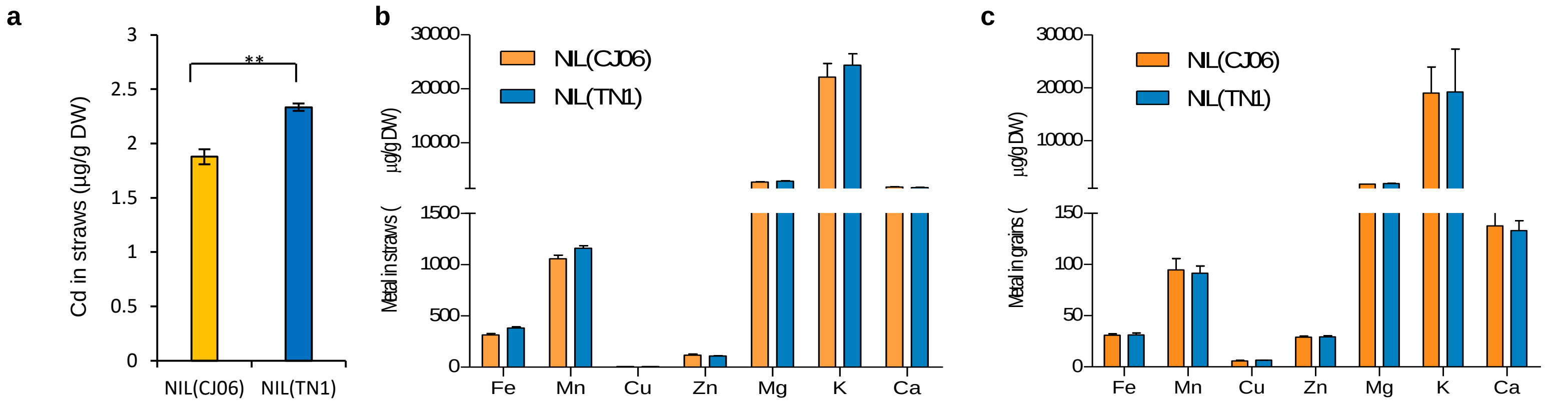

Supplementary Figure 5. Metal accumulation in rice straws and grains of soil-grown NIL lines. NIL(TN1) and NIL(CJ06) plants were grown in heavy metal polluted paddy field until ripening. (a) Cd accumulation in straws, (b-c) essential metal (mineral nutrients) accumulation in straws (b) and grains (c). Data are mean ± SD, n= 5. Significant differences were determined by Student's *t*-test (**P < 0.01)

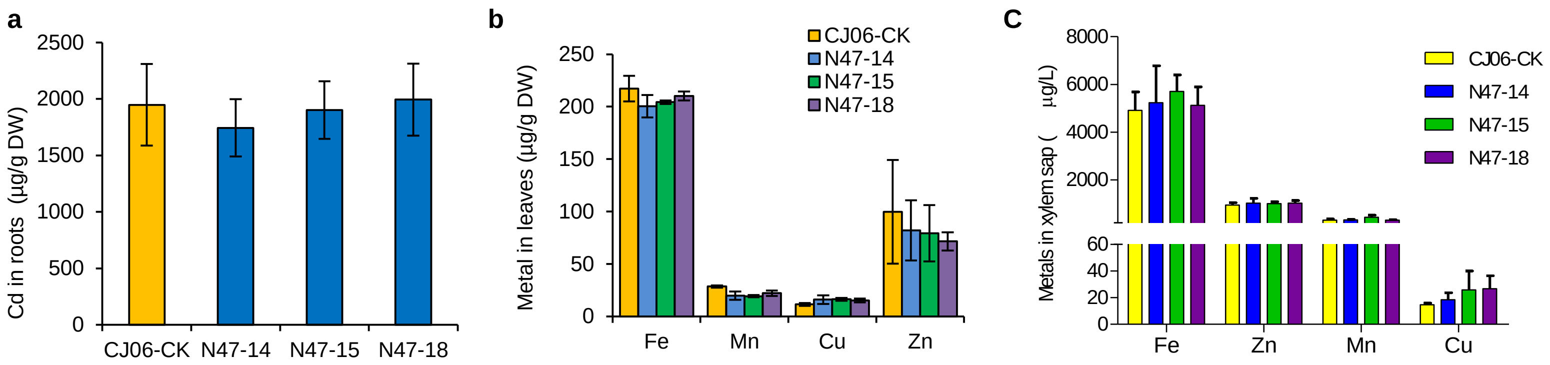

Supplementary Figure 6. **Metal accumulation in the *CAL1* complementation lines.** The identified complementation lines N47-14, N47-15 and N47-18 (Supplementary Figure 3d) were germinated and grown in hydroponics for 2 weeks before treatment with 10uM Cd for 7days. CJ06-CK represent transgene-negative controls. The plants were then sampled to determine Cd accumulation (a) in roots, metals accumulation in leaves (b) and xylem sap (c). Data are mean ± SD, n = 4.

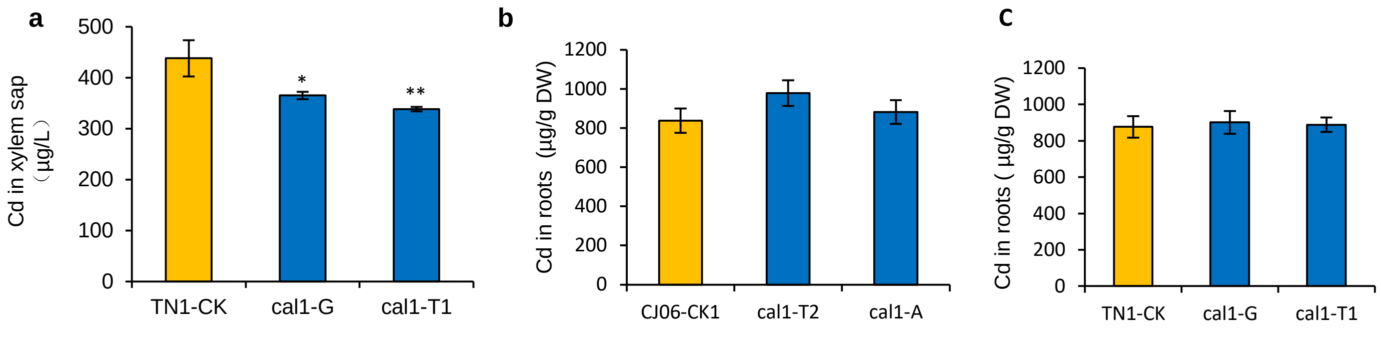

Supplementary Figure 7. **Cd accumulation in *cal1* mutant roots.** (a-c) Two weeks old seedlings grown in hydroponics were exposed to 10 μM Cd for 7days. Cd contents in xylem sap (a) and roots (b, c) of *cal1* mutants were determined. TN1-CK and CJ06-CK1 represent transgene-negative controls. Data are mean ± SD, n = 3. Significant differences were determined by Student's t-test (*$P < 0.05$, **$P < 0.01$).

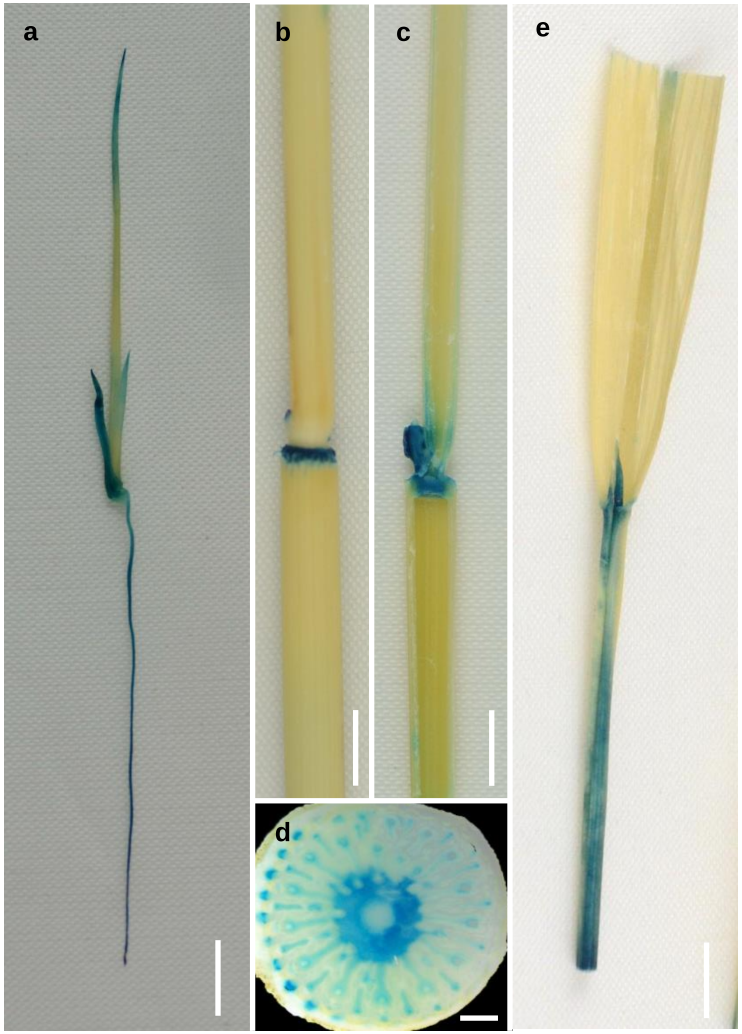

Supplementary Figure 8. **Histochemical localization of GUS activity in transgenic plants expressing the GUS reporter gene under the control of the pro*CAL1*^{TN1} promoter.** (a) One week old whole-mount seedling. (b, c) Elongated internodes, node I (b) and the longitudinal section of these tissues (c). (d) Cross section of node I at 14 weeks of age. (e) Flag leaf sheath and flag leaf blade. Bars = 1 cm in (a-c, e), 100 µm in d.

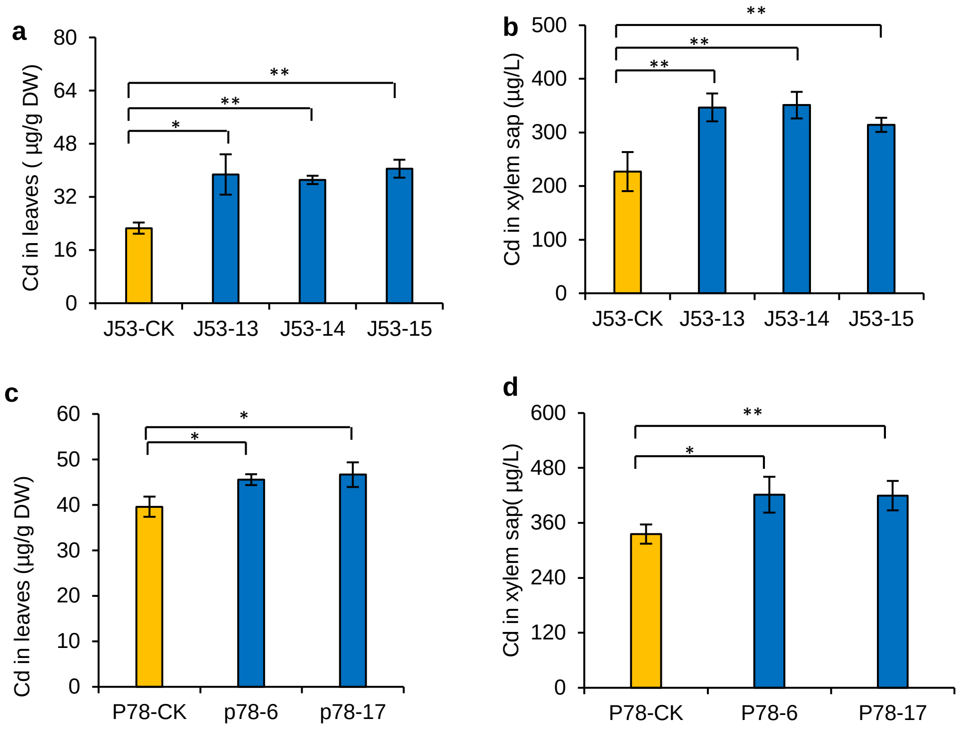

Supplementary Figure 9. The *CAL1-mRFP* fusion proteins are functional. Transgenic plants harboring 35S::*CAL1-mRFP* (a, b) or pro*CAL1*^{TN1}::*CAL1-mRFP* (c, d) in CJ06 genetic background were grown in hydroponics for two weeks before exposure to 10 µM Cd for 7days. Cd contents in leaves (a, c) or xylem sap (b, d)were determined. J53-CK represents transgenic plants not harboring the construct 35S::*CAL1-mRFP*. While P78-CK represents plants not harboring the construct pro*CAL1*^{TN1}::*CAL1-mRFP.* Data are mean ± SD, n= 3. Significant differences were determined by Student's *t*-test (**P* < 0.05, ***P* < 0.01).

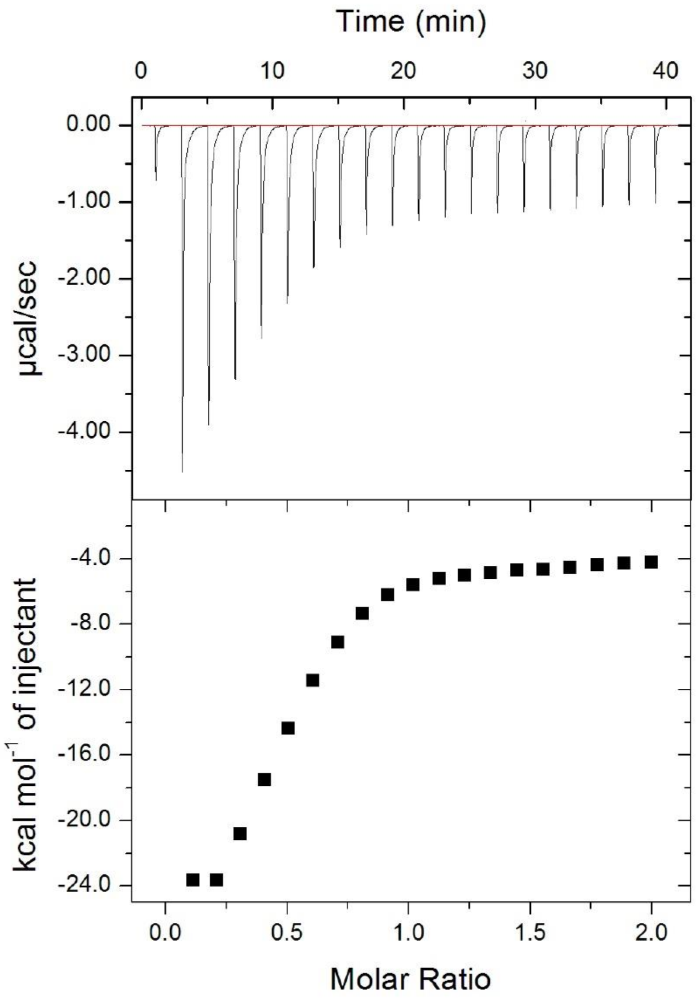

Supplementary Figure 10. Determination of ΔSPCAL1's binding affinity to Cd using isothermal titration calorimetry at pH 7.5 as described in Method.

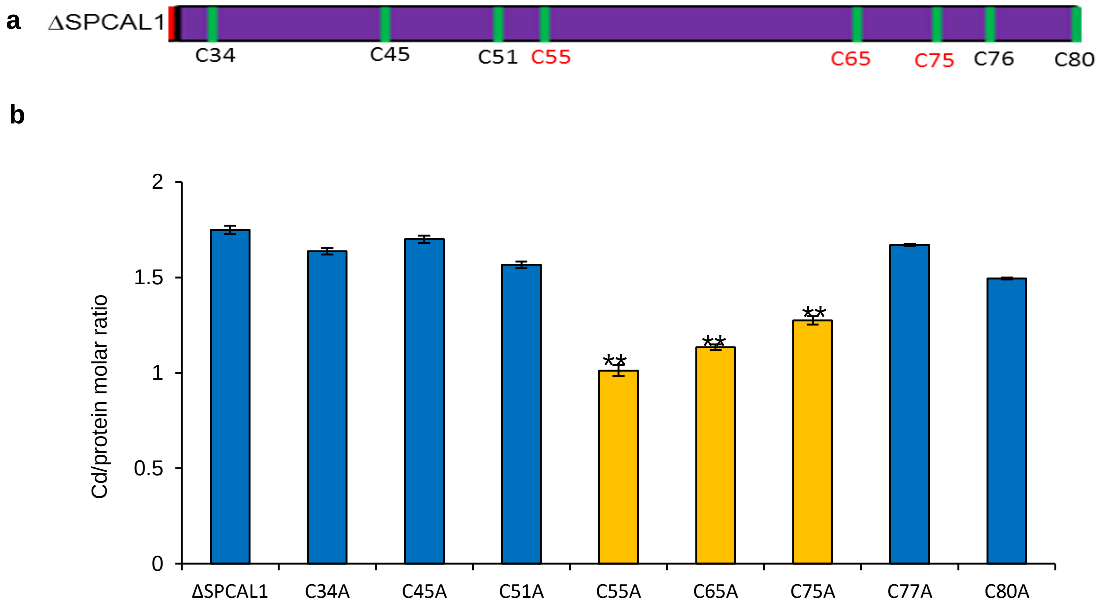

Supplementary Figure 11. Cd-binding assay for ΔSPCAL1 variants derived from site-specific mutagenesis. (a) Structural model of the ΔSPCAL1 protein. Green bars indicate cysteine residues at corresponding sites along protein sequences. (b) ΔSPCAL1 variants were isolated and incubated with 100 µM Cd at pH 7.5, as described in Method. Data are mean ± SD, n= 3. Significant differences were determined by Student's *t*-test (***P* < 0.01).

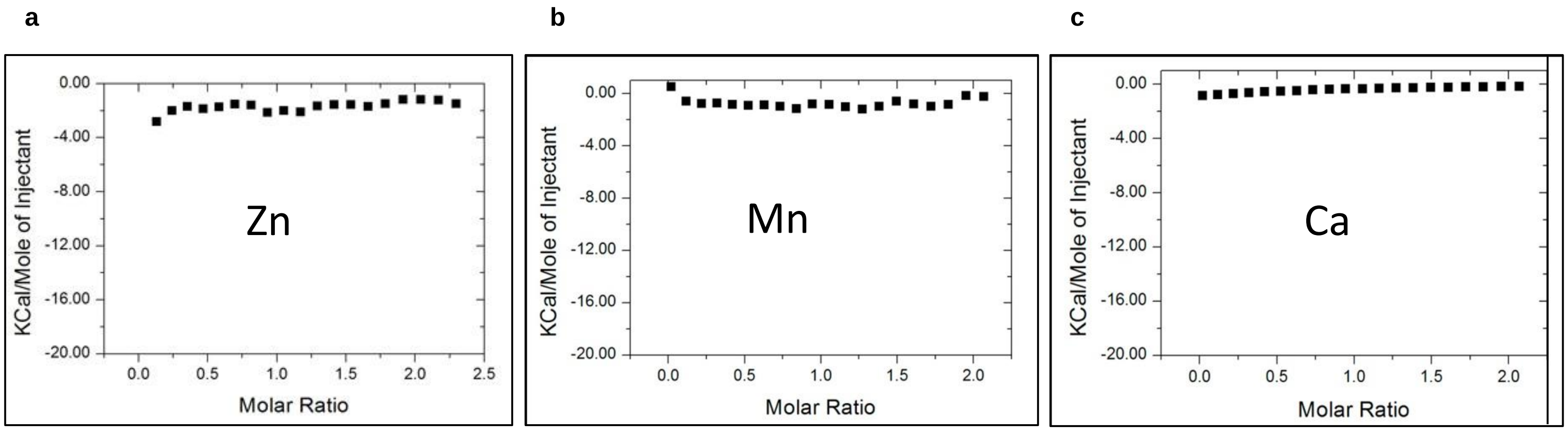

Supplementary Figure 12. **Metal-binding affinity of ∆SPCAL1 assayed by isothermal titration calorimetry at pH 7.5.**

(a) Zn-binding affinity of ∆SPCAL1. (b) Mn-binding affinity of ∆SPCAL1. (c) Ca-binding affinity of ∆SPCAL1.

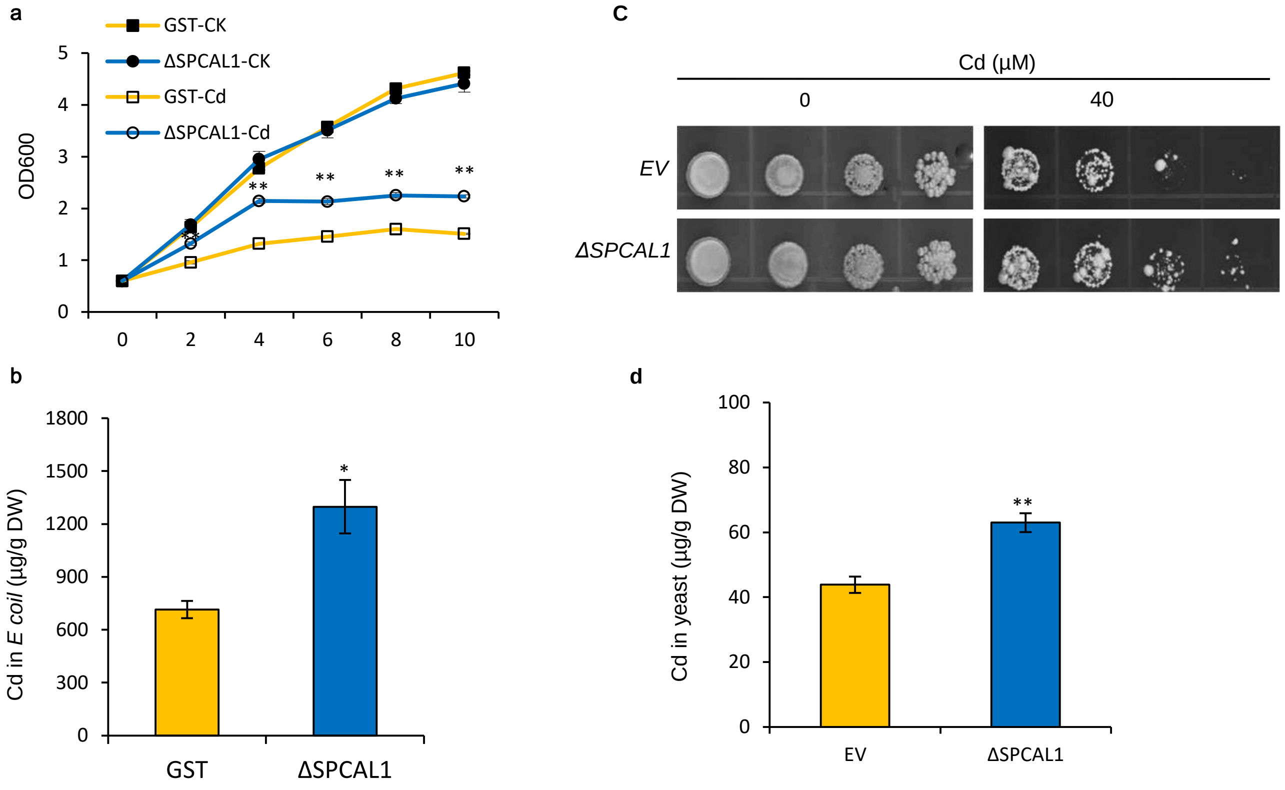

Supplementary Figure 13. **CAL1 enhanced Cd tolerance in *Escherichia coli* and yeast.** (a) Growth curves for *Escherichia coli* strains expressing ΔSPCAL1. Blue lines indicate GST-ΔSPCAL1 fusion in which the secretion signal peptide of CAL1 was deleted to simulate mature protein. Yellow lines indicate the GST control. Close box and circle represent control condition without Cd (CK); Open box and circle represent treatment with 200 μM Cd (Cd). (b) Cd concentration in *Escherichia coli* strains from (a) incubated for 4 hours was determined by ICP-MS. (c) Δ*yap1*, a Cd-sensitive yeast mutant in which activation of *YCF1* expression was impaired, was transformed with the blank vector (EV) or *ΔSPCAL1.* The transformants were plated and grown in the presence of 0 or 40 μM Cd at 30℃ for 7 days before imaging. Galactose was added to activate gene expression. (d) Yeast strains from (c) were incubated with 20 μM Cd in SD medium for 2 hours. Cd concentrations in the harvested cells were determined by ICP-MS. Data are mean ± SD, n= 3 in (a, b, d). Significant differences were determined by Student's *t*-test (***P* < 0.01).

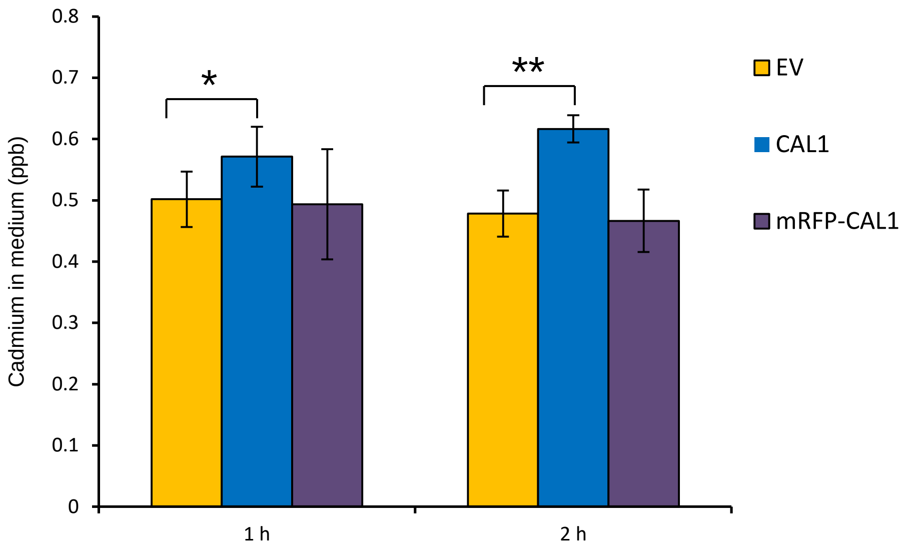

Supplementary Figure 14. Cd extrusion enhanced in yeast expressing CAL1. Yeast transformants were grown to OD600 of 1.0 and treated with 20 μM Cd for 2 hours. Cells were collected and washed twice with ultrapure (>18 MΩ cm^{-1}) water, 25 mM $CaCl_2$ (pH 5.0) and ultrapure water again in succession. The washed cells were resuspended in fresh liquid SD medium to OD600 of 1.5, then incubated for 1 or 2 hours before removal of cells by centrifuge (12000 RPM for 2 min). Cd content in the collected supernatant was determined by ICP-MS. EV, empty vector, CAL1 and mRFP-CAL1 represent the full length or non-secreted form of CAL1 protein, respectively. Data are mean ± SD, n = 8. Significant differences were determined by Student's *t*-test (*$P < 0.05$, **$P < 0.01$).

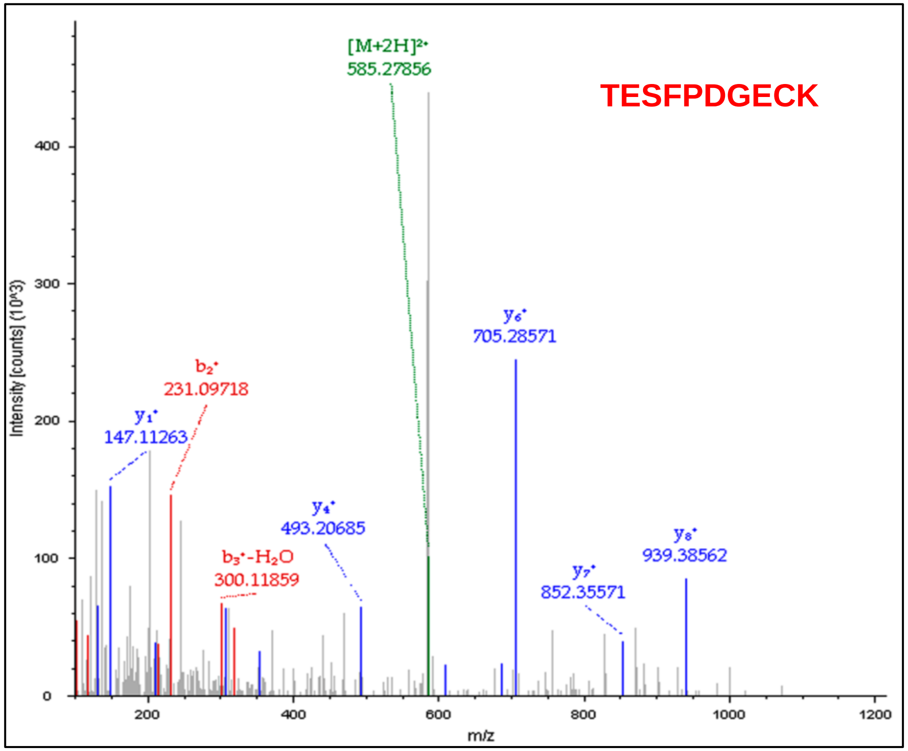

Supplementary Figure 15. Detection of the CAL1 fragment in xylem sap by Electrospray Ionization Mass Spectrometry (ESI-MS). Rice seedlings of the *CAL1*-complementation lines N47 were grown for 21 days in hydroponic solution supplemented with 5 µM Cd, then xylem sap was sampled for ESI-MS assay. The sequence of the detected CAL1 fragment was displayed in red at the upper right corner.

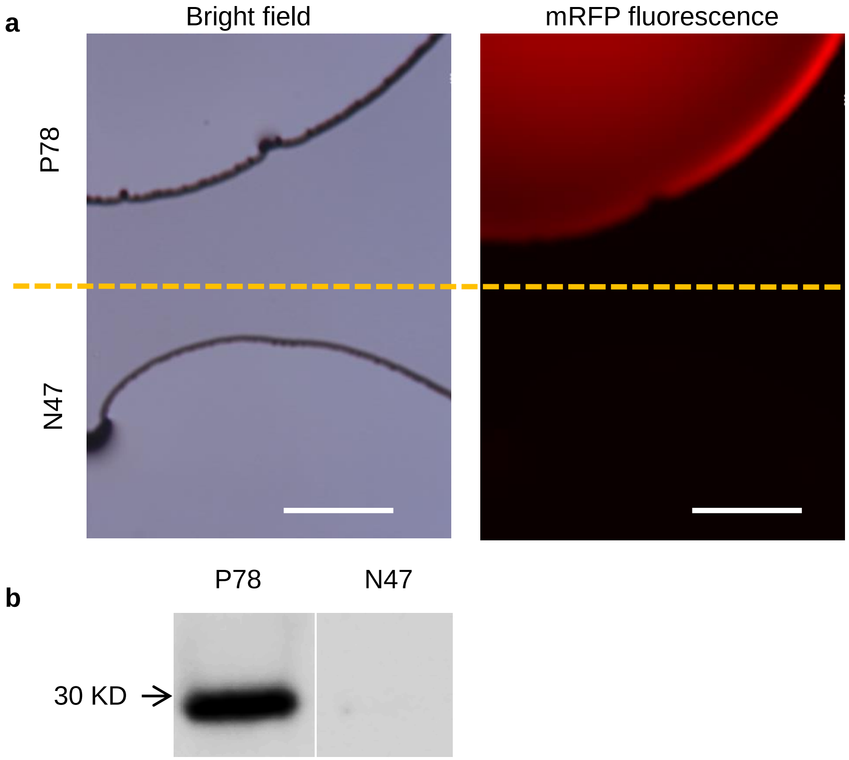

Supplementary Figure 16. Detection of CAL1 in guttation fluid from intact rice leaves. Transgenic plants P78 harboring the construct proCAL1^{TN1}::CAL1-mRFP, or N47 harboring the complementation construct proCAL1^{TN1}::CAL1 were used. Leaf guttation fluid was collected with a micropipette in the morning. Sampled leaf guttation fluid was then subjected to assay of fluorescence imaging (Leica, DM6000B) and Western blotting. **(a)** Fluorescence images of guttation fluid from P78 or N47. Bar = 200 µm. **(b)** Western blot detection of CAL1 in guttation fluid from P78 and N47 using anti-RFP antibody.

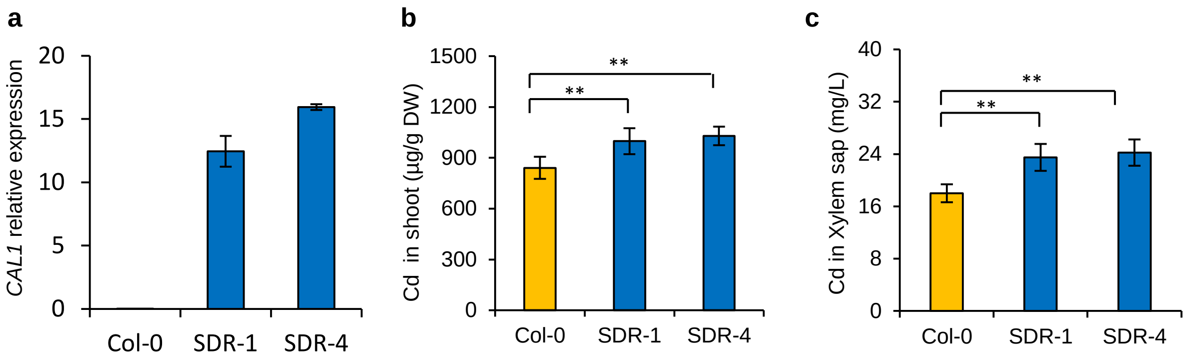

Supplementary Figure 17. Ectopic expression of *CAL1* increased Cd contents in *Arabidopsis* shoots and xylem sap. Four weeks old of 35S::*CAL1-mRFP* transgenic plants were exposed to 10 µM Cd for 3 days. Heterologous overexpression of *CAL1* (a) increased Cd contents in *Arabidopsis* shoots (b) and xylem sap (c) . *Actin2* was used as an internal control in (a). Data are mean ± SD, n= 3 in (a-c). Significant differences were determined by Student's *t*-test (***P* < 0.01).

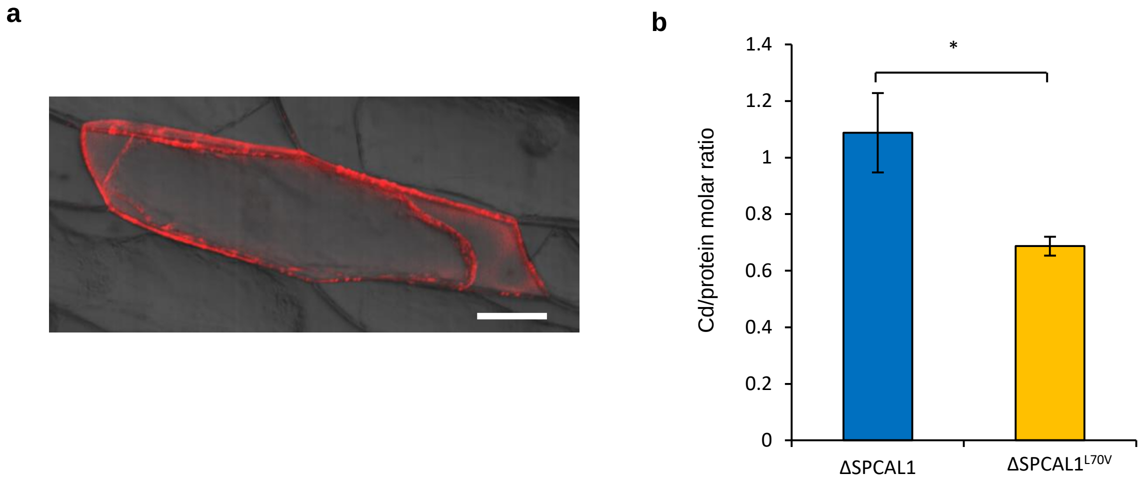

Supplementary Figure 18. Functional characterization of CAL1^{L70V}. (a) Onion epidermal cells transformed with the 35S::*CAL1*^{L70V}*-mRFP* construct were incubated in 40% sucrose to induce plasmolysis and then imaged by confocal microscopy. Bar = 20 µm. (b) Different forms of CAL1 recombinant proteins were isolated and incubated with 100 µM Cd at pH = 7.5, as described in Material and Method. Data are mean $\pm$ SD, n = 3. Significant differences were determined by Student's *t*-test (*$P < 0.05$).

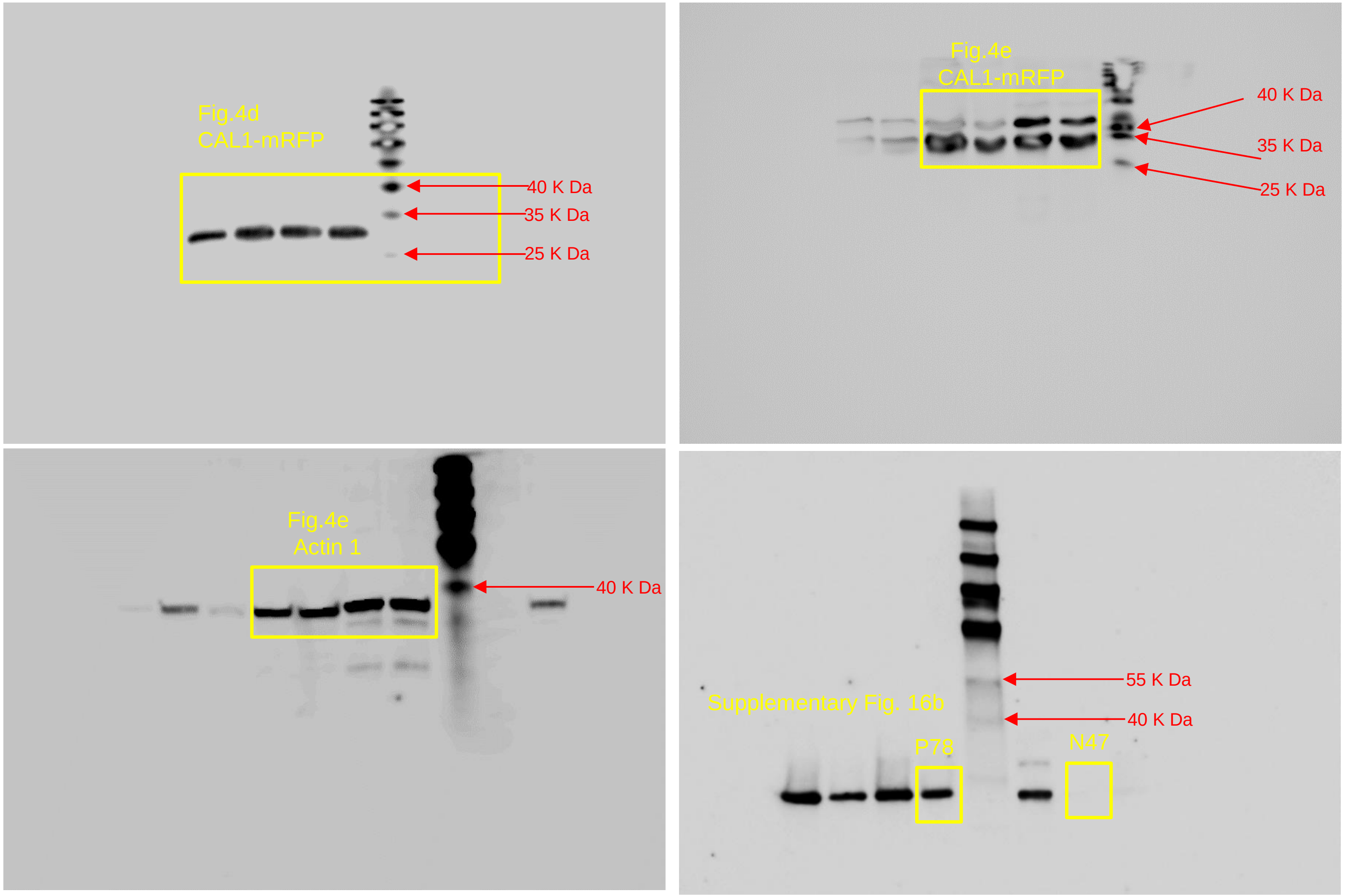

Supplementary Figure 19 Full uncropped versions of western blot images. Full uncropped versions of western blot images shown in Fig. 4d-e and Supplementary Fig. 16b. The yellow rectangles indicate the cropped area. The red arrows indicate the molecular markers.

Supplementary Table 1. Index to the rice accessions used for Ionomic screening

Serial No.	Accession	Serial No.	Accession	Serial No.	Accession	Serial No.	Accession	Serial No.	Accession	Serial No.	Accession
1	HG2	37	ZL1-1	73	LSZ	109	WZD	153	ZH	189	SY352
2	LGT83	38	YLCH	74	TDLD	110	XBN	154	MZ	190	CYPRES
3	EJN1	39	SF101	75	TZXX2	111	MBWX	155	LGZ	191	AUS43
4	CY1B	40	CDA3	76	DY5	112	QYX	156	SRZ	192	ITA182
5	AJNT	41	GJ73	77	YMZG	113	ZG	157	JYZ	193	TN1
6	L301B	42	JBL	78	MGZ	114	MWZ	158	ZST	194	CJ06
7	GLA4	43	TSN	79	MJD2	115	MMG	159	HGZ	195	YG
8	XAZ10	44	GC2	80	AM	116	CGN	160	MDZ	196	SKC1
9	JNT43B	45	XGN	81	HBYY	117	ZN	161	SCGJMD	197	HQ0547
10	XZX7	46	HK3	82	NTH	118	QK	162	LMD	198	HQ0345
11	80B	47	TZZL1	83	WLX	119	BZN	163	LSD	199	2S97
12	GLA15-	48	TQXH	84	ESN	120	WJHG	164	YQN	200	NIPPO
13	ZZB	49	HSGZ	85	JFX	121	NGG	165	NFZ	201	TP309
14	BX123B	50	ATG151	86	CSL	122	MWGN	166	QQB	202	ZH11
15	HMD	51	XWX3	87	HKZN	123	HL	167	BMZ	203	HD
16	BX-7B	52	TZ65	88	NTGJ	124	ZM	168	JXZ	204	CJSD
17	JNTB	53	ZSNH6	89	QTBG	125	QTG	169	PA64	205	UN
18	FNZP	54	JY1	90	HXLC	126	PWS	170	ZGN	206	LLXBM
19	ZS97B	55	CLSJ	91	HMD	127	YZX	171	MGN	207	LLNZ
20	GZS97B	56	GHH	92	LHZ	128	GKXN	172	LZN	208	CDJZ
21	QSA16B	57	MM	93	BKZH	129	WXN	173	ZJG	209	FHD
22	LMB	58	XS115	94	AHC	130	XMX	174	BKSG	210	TDYSX
23	IR661-1	59	SBL	95	HMK	131	XBM	175	QSD	211	HSN
24	NJ11	60	JD1	96	SB70	140	QXBDD	176	LCH	212	ZJNG
25	WG	61	XG	97	SJG	141	LLDBM	177	TQ	213	HMDZ
26	G630	62	DDLD	98	LXG	142	TSCXD	178	CMN	214	TGG
27	DR409B	63	ZH8	99	XN	143	MZW	179	BG951	215	QKG
28	LG287	64	LS1	100	NKN	144	HSB	180	IAC1300	216	BGZ
29	HH628	65	BWB1	101	YKN	145	XFLF	181	IRAT10	217	BJG2
30	JHB	66	AMK	102	TGW	146	YZL	182	IRAT216	218	YGZ
31	88B	67	DTWX	103	XECT	147	YMTK	183	Kasalath	219	ZG
32	G154	68	YD2	104	XD	148	WND	184	93-11	220	MDZ
33	NH21	69	ZD5	105	MHN	149	XHM	185	RBQ		
34	XH91269	70	ZX232	106	XHG	150	TTND	186	LEMONT		
35	LHMH	71	G87-304	107	ZN4	151	GLW	187	ITA408		
36	LCH	72	HW1	108	LHD	152	HTH	188	NK57		

Supplementary Table 2. Quantitative trait loci (QTLs) for Cd accumulation in rice leaf

Plant part	QTLs	Chr	Marker interval	LOD [a]	% of variance explained [b]	Additive effect [c]
leaves	qLCd2	2	RM324-RM5472	2.84	13.1%	12.792
	qLCd4	4	RM3276-RM225	2.15	11.2%	10.122
	qLCd11	11	RM21-RM3428	2.07	11.0%	-7.8390

Rice seeding were grown in hydroponics as described in Methods, then exposed to 10 µM Cd for 7 days before sampling for data collection and QTL analysis. a: Maximum LOD score at the QTL position, b: Percent of phenotypic variance explained by QTL, c: A positive value indicates that the allele from TN1 increases the phenotypic value.

Supplementary Table 3. Annotation of candidate genes in the QTL region of chromosome 2

Locus ID	Annotation
Os02g0628600 (ARF)	Similar to auxin response factor 8
Os02g0629200 (MIP)	Major intrinsic protein (MIP) superfamily
Os02g0630000 (F-box)	F-box domain, cyclin-like domain containing protein
Os02g0629800 (CAL1)	Similar to defensin precursor

Supplementary Table 4 Cd-binding affinity of ΔSPCAL1 and the related mutants assayed by isothermal titration calorimetry

Mutants	Dissociation constant/KD(μM)
ΔSPCAL1	53.7 ± 4.6
C55A	197.2 ± 15.7 **
C65A	204.1 ± 21.3 **
C75A	96 ± 8.5 *

The wild type ΔSPCAL1 and the three variants with significantly decreased Cd binding ability (Supplementary Figure 11b) were used for ITC analysis, at pH 7.5. Data are mean ± SD, n= 3. Significant differences were determined by Student's *t*-test (**P* < 0.05 ***P* < 0.01).

Supplementary Table 5. Primers used in this study

Primer	Forward primer	Reverse primer
**Map based cloning of CAL1 primer**		
RM324	CTGATTCCACACACTTGTGC	GATTCCACGTCAGGATCTTC
B3	GGAGAGTGAGAGCCAAATGA	GCCTTAATAGTCGGCTTAGC
EcoR1	TGAGTCATATTCAGTCAACC	ACAGCAAGATCGTGGTATTA
RM13628	TATGCCACGAATGACCCTAACC	CTCCATATGCAGCGACAATCG
X3	CAGCTGACCTAACTACGCCC	GCCAAGGATTCTTCTGTGCA
C1	GGTGAGTCTAGGCCAATATG	AGAGAACCAAGAGGGATTGC
C2	GAGCAGGTACAATAGCAGAC	AGACAAGCATGCAGAAAGTA
D1	ATGTGGGCCCCGCTATTTTA	GTAAGCACACAGATTCATCA
F1	GAGACGTCTCCGACAACACC	CAGCATTTAGCGCCTAGTTG
H10	ACCACGCATCAGTTGCACGC	CGGGTCTCACGCAATTGTTC
M6	GTAATGGTTTAATGCAATGC	ACTGAGGTGGTCAATGAGTT
H21	AGTACTTGTACTTGCAAAGG	TATATGTTTGCTAACGATCG
RM13652	TTAAGTTTCGGCTCCTTCACTCG	CACACATATGCATCCACAGTTCC
E6	CTGTCCGTGCACGACGTATT	GAGGTGGATTTGGAACTGTG
Nhe1	GACATCACAGACGCGGCAAT	GCTTTTCCCCAATTTGACGT
RM263	CCCAGGCTAGCTCATGAACC	GCTACGTTTGAGCTACCACG
**Real Time PCR**		
CAL1	AGTCGCGTGTTCTCCTTTGT	CATGACAGCAGCTTGCAAAT
HistonH3	GGTCAACTTGTTGATTCCCCTCT	AACCGCAAAATCCAAAGAACG
Actin 1	TCCATCTTGGCATCTCTCAG	GTACCCGCATCAGGCATCTG
ARF	CCATCAACCTCTCGTCTTTCTC	GTGGTTGTGATGGTAATGGTG
MIP	CTGTCCAGCACTAGATCAACG	TGGCGGACATAGTTCAAAGG
F-box	TTCCGCTACGCTTGGTTC	ACAAAACTGAACTTGTTACACGG
Actin 2	AGGTATCGCTGACCGTATGAG	CATCTGCTGGAATGTGCTGA
**Transgenic plant**		
ProCAL1	AAGCTTCCCATGTGGGCCCCGCTATT	GGATCCCTGTACCGGCGACTCGAACT
sgR-CAL1	TGGCGGGGACGACCAAGGTGGCGG	AAACCCGCCACCTTGGTCGTCCCC
HB-CAL1	AAGCTTATGTGGGCCCCGCTATTTTA	GAGCTCGTAAGCACACAGATTCATCA
HB-F-box	AAGCTTGAGACGTCTCCGACAACACC	GAGCTCCAGCATTTAGCGCCTAGTTG
HB-ARF	AAGCTTGGTGAGTCTAGGCCAATATG	GGTACCAGAGAACCAAGAGGGATTGC
HB-MIP	GGATCCGATGACAAGATGAAGGACCT	TCTAGACTCCACAGACAAGCATGCAG
**CAL1 subcellular localization**		
5’CAL1-RFP	GGATCCATGGCTCCGTCTCGTCGCAT	ACTAGTGCAGACCTTCTTGCAGAAGC
3’CAL1-RFP	TCTAGAATGGCTCCGTCTCGTCGCAT	GGATCCCTAGCAGACCTTCTTGCAGA
RFP	ACTAGTATGGCCTCCTCCGAGGACGT	GAGCTCTTAGGCGCCGGTGGAGTGGC
**Prokaryotic expression of CAL1**		
ΔSPCAL1	GGATCCAGGCACTGCCTGTCGCAGAG	GAATTCCTAGCAGACCTTCTTGCAGA
TF-CAL1	GGATCCATGGCTCCGTCTCGTCGCAT	GAATTCCTAGCAGACCTTCTTGCAGA
**Transgenic plants screening**		
HYG	CTTCTGCGGGCGATTTGTGT	TTATCGGCACTTTGCATCGG

Supplementary Table 5 continued

Primer	Forward primer	Reverse primer
	Site-specific mutagenesis	
C34A	GGATCCAGGCACGCCCTGTCGCAGAGCCAC	GGCGTGCCTGGATCCCTCGAGGGTACCGAG
C45A	TTCAAGGGCATGGCCGTGAGCAGCAACAAC	GGCCATGCCCTTGAACCTGTGGCTCTGCGA
C51A	AGCAGCAACAACGCCGCCAACGTGTGCAGG	GGCGTTGTTGCTGCTCACGCACATGCCCTT
C55A	TGCGCCAACGTGGCCAGGACGGAGAGCTTC	GGCCACGTTGGCGCAGTTGTTGCTGCTCAC
C65A	CCCGACGGCGAGGCCAAGTCGCACGGCCTC	GGCCTCGCCGTCGGGGAAGCTCTCCGTCCT
C75A	CTCGAGCGCAAGGCCTTCTGCAAGAAGGTC	GGCCTTGCGCTCGAGGCCGTGCGACTTGCA
C76A	CGCAAGTGCTTCGCCAAGAAGGTCTGCTAG	GGCGAAGCACTTGCGCTCGAGGCCGTGCGA
C80A	TGCAAGAAGGTCGCCTAGGAATTCAAGCTT	GGCGACCTTCTTGCAGAAGCACTTGCGCTC
